# Task Relevance Modulates Somatosensory Awareness Depending on Stimulus Intensity

**DOI:** 10.1111/ejn.70262

**Published:** 2025-09-25

**Authors:** Alice Giorgi, Erica Bonomi, Enrico Salemi, Davide Albertini, Flavia Maria Zauli, Ezequiel Pablo Mikulan, Andrea Pigorini, Pietro Avanzini, Maria Del Vecchio

**Affiliations:** ^1^ Neuroscience Institute National Research Council of Italy Rome Italy; ^2^ Department of Medicine and Surgery University of Parma Parma Italy; ^3^ Department of Biomedical and Clinical Sciences University of Milan Milan Italy; ^4^ Department of Health Sciences University of Milan Milan Italy; ^5^ Department of Biomedical, Surgical and Dental Sciences University of Milan Milan Italy; ^6^ UOC Maxillo‐Facial Surgery and Dentistry Fondazione IRCCS Cà Granda, Ospedale Maggiore Policlinico Milan Italy

**Keywords:** EEG, P3b, SAN, SEPs, Somatosensation, tactile awareness

## Abstract

Recent studies on somatosensory evoked potentials (SEPs) have proposed that the P3b component reflects higher order postperceptual processes, such as stimulus reporting and task relevance, whereas somatosensory awareness negativity (SAN) is more directly associated with somatosensory awareness. Despite growing evidence supporting this functional distinction, the omission of no‐report conditions and controls for task demands hindered a clear separation between report‐ and task‐related processes from those linked with somatosensory awareness. Here, we designed a simple experimental procedure that varied stimulus amplitude (set at individual sensory or motor thresholds) and task relevance (no‐report stimulation, report + task‐relevant stimuli, report + task‐irrelevant stimuli) with two main objectives: first, to verify that P3b appears only when a report is required, being abolished in a no‐report condition, and second, to determine the extent to which SAN is modulated by task requirements and stimulation amplitude. Our results closely link P3b with task relevance and show that SAN is indeed modulated by task relevance but only when stimuli are delivered at the verge of detection. In other words, task relevance influences conscious perception through an enhancement of the associated neural responses but only for stimuli challenging to detect. Overall, our findings provide evidence that P3b is closely associated with task relevance, remaining out of the correlates of sensory awareness. In parallel, SAN modulations serve as a useful proxy for awareness in experimental manipulations involving attentional factors but only when stimuli are delivered near the sensory threshold.

## Graphical abstract

The study shows that the P3b component of somatosensory evoked potentials (SEPs) is tight to task relevance, whereas somatosensory awareness negativity (SAN) is influenced by task demands but only for stimuli near the sensory threshold, reflecting enhanced awareness through selective attention. These findings clarify the distinct roles of P3b and SAN in processing task relevance and sensory awareness.

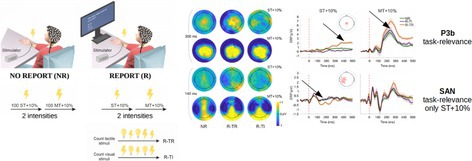

**Abbreviations**
EEG: electroencephalography
ERPs: event‐related potentials
PAN: perceptual awareness negativity
SAN: somatosensory awareness negativity
SEPs: somatosensory evoked potentials
VAN: visual awareness negativity

## 1 Introduction

Over the past century, physiologists have leveraged the temporal resolution of electroencephalography (EEG) to track brain activity associated with somatosensory processing. Together with the understanding of the cortical processes subtending tactile encoding (Dijkerman and de Haan 2007; Sathian 2016), attention has been paid to the mechanism underlying the conscious perception of tactile stimuli (de Haan and Dijkerman 2020; Hirvonen and Palva 2016; Palva et al. 2005; Pleger and Villringer 2013; Schröder et al. 2019). These aspects have been investigated, questioning possible modulations of somatosensory evoked potentials (SEPs) elicited by experimental manipulations, underlying a dichotomy between their early and late components. Early components, like N20 and P50, likely originate from primary somatosensory cortex (Allison et al. 1989; Forss et al. 1994; Hämäläinen et al. 1990) and are associated with stimuli features such as intensity (Forschack et al. 2020) and duration (Spackman et al. 2006); in turn, components with peaks occurring later than 100 ms are reported to be modulated by higher‐order functions such as spatial attention (e.g., P100; Eimer and Forster 2003), stimulus awareness, reporting, and task relevance (e.g., N140 and P3b; Dembski et al. 2021).

Within these, the distinction between the neural underpinnings underlying sensory awareness and task relevance has been blurred by confounds in the experimental procedures, thereby limiting the possibility to assess the strength of their interaction. Indeed, the neural correlates of sensory awareness have been largely investigated with tasks that implicitly assess perceptual attributes (e.g., subjects were required to identify or ignore specific categories of stimuli), or more commonly, subjects had to explicitly report whether a stimulus—delivered at the threshold of detection—was perceived (Tsuchiya et al. 2015). This type of paradigm poses a problem, in that all subjects receive an assignment that incorporates both task relevance and/or reporting, resulting in the impossibility to isolate the correlates specifically sustaining somatosensory perception. Despite these experimental pitfalls, however, a growing consensus supports the idea that SAN (e.g., the somatosensory awareness negativity, which occurs between 125 and180 ms after stimulus delivery) represents the scalp neural fingerprint of somatosensory awareness, whereas P3b is, in turn, closely associated with postperceptual phenomena such as task relevance, reporting, and working memory (see Dembski et al. 2021).

Nevertheless, the aspects of postperceptual phenomena that the P3b encodes and how contextual information (e.g., instructions given to the subjects) interact with sensory awareness, possibly sustaining it, remain unresolved because of the lack of a true basal condition for comparison (e.g., a train of simple, supra‐threshold, perceivable, and task‐irrelevant stimuli (Avanzini et al. 2018; Del Vecchio, Caruana, et al. 2019; Del Vecchio, Fossataro, et al. 2021). Regarding the possibility of assessing whether task relevance interacts with sensory awareness, previous studies (Pitts, Metzler, et al. 2014; Shafto and Pitts 2015; Schelonka et al. 2017) have highlighted that VAN—the visual homologue for SAN—has significantly greater amplitude when stimuli are task relevant. However, task relevance is rarely declined across experimental conditions in a controlled manner, which limits our ability to fully understand the strength of the relationship between this factor and awareness. To overcome this limitation, we designed an experimental procedure aimed at providing a quantitative assessment of the effects of task relevance—independent of report‐related processes—on the P3b and SAN components. This was achieved by delivering tactile stimuli that were either task‐relevant (in the report condition) or task‐irrelevant (in both the report and no‐report conditions). Additionally, all the stimuli were administered at two different amplitude conditions (e.g., at sensory and motor thresholds [ST and MT]), based on the hypothesis that attentional mechanisms are more effective when stimuli are presented at lower intensities. Our paradigm is specifically designed to investigate the relative contributions of report and task relevance to SEPs, incorporating a true baseline condition—featuring clearly detectable, task‐irrelevant stimuli presented without the requirement to report. Our findings associate the P3b component with task relevance and demonstrate that SAN is influenced by task relevance only when stimuli are presented at the threshold of detection. This provides evidence that perception is enhanced by selective attention only when it is needed.

## 2 Materials and Methods

### 2.1 Participants

We recruited 30 right‐handed participants (age: 24 ± 3, 11 males). All participants were naive to the purpose of the experiment and had normal or corrected‐to‐normal vision, with no history of psychiatric and neurological disorders. The local ethical committee approved the study (CNR Commission for Ethics and Integrity in Research n. 0065527/2019), which was conducted according to the principles expressed in the Declaration of Helsinki. Each participant provided written informed consent before the experiment.

### 2.2 Experimental Procedure

#### 2.2.1 Individual ST Identification

The median nerve stimulation was delivered using a Digitimer DS7A, which conveyed the electrical signal to two electrodes placed at the right wrist. Stimulations, all lasting 0.2 ms, were delivered at two distinct, spaced‐apart amplitudes, that is, the MT and the ST. First, MT was determined by stimulating at an amplitude high enough to provoke an observable thumb twitch. Subsequently, to identify ST, participants underwent an adaptive staircase procedure. Specifically, they listened to two auditory cues and, after hearing both, indicated whether the tactile stimulation occurred after the first or the second cue. If the stimulation was correctly identified three consecutive times, the amplitude was progressively lowered by 10%; this continued until participants made mistakes or missed the stimulation. In these latter cases, the last tested amplitude was first increased by 5% and then lowered again by 3% after they provided three correct answers. Finally, the amplitude was further increased by 1%; after this level, the whole procedure continued with lowering and increasing the amplitude by 0.5% six times. The ST was then calculated by averaging the amplitude levels during these final six steps (Harris et al. 2007). Finally, stimulation intensities were set at MT and ST, both increased by 10% (i.e., MT + 10% and ST + 10%, respectively) to avoid habituation effects (Klingner et al. 2011).

#### 2.2.2 Stimuli

The experimental paradigm comprises two blocks: *No Report* (NR) and *Report* (R). During the NR condition, participants were instructed to passively perceive two trains of 100 tactile stimulations (duration = 0.2 ms), spaced by a random time ranging from 1 to 2 s. One first train was administered at ST + 10%), and a second train was delivered at MT + 10%. The R block contained 40 series of unimodal stimuli, except for two subjects who completed 28 and 32 series of stimulation, respectively. These could be either visual (i.e., a flash delivered on the screen) or tactile, organized in 20 trains at ST + 10% and 20 trains at MT + 10% presented in a random order. For each sequence, visual and tactile stimuli are presented in a variable number (6 ± 2 for visual stimuli, 7 ± 3 for tactile ones). In the Report section, participants were asked to follow instructions on the screen indicating whether they should count visual (report task‐irrelevant condition, R‐TI) or tactile stimuli (report‐task‐relevant, R‐TR) in the subsequent train. At the end of each series, three numbers with an associated letter appeared on the screen; subjects were then asked to press the letter on the keyboard that represented the correct number of counted stimulations.

### 2.3 EEG Data Collection

EEG data were continuously recorded at a sampling rate of 500 Hz using a vertex reference. Recordings were made with the 128‐channel Geodesic EEG System (Electrical Geodesics Inc.) and the HydroCel Geodesic Sensor Net, which includes 19 electrode sensors (AgCl‐coated electrodes) positioned in a geodesic pattern corresponding to the 10–20 system locations. To ensure consistent electrode placement across participants, the Sensor Net was aligned with skull landmarks (nasion, vertex, and preauricular points). We used the Net Amps300 high‐input impedance amplifier. Low‐noise EEG data were obtained, guaranteeing sensor‐skin impedances below 50 kΩ except for the reference one, which was kept below 10 kΩ.

### 2.4 Preprocessing

EEG data were imported into MATLAB for analysis using EEGLAB v2021.0 (Delorme and Makeig 2004). To reduce contamination from muscular artefacts, we excluded the outermost belt of electrodes of the sensor net—19 peripheral channels located on the cheeks and nape (Michel et al. 2004). Additional channels with abnormal activity were identified through visual inspection and subsequently interpolated. Gross artefacts involving all electrodes (e.g., resulting from abrupt head or body movements) were then removed, and subsequently, to identify artefacts related to eye movement, muscle activity, and residual channel noise, we computed the independent component analysis (ICA) on the EEG principal components that accounted for 99% of the data variance. To this aim, data were first notch filtered ([48, 52] Hz) and band‐pass filtered ([2, 100] Hz) (Klug and Gramann 2021), segmented in epochs around the stimulus delivery ([−0.3, 0.7 ms]) and visually inspected to remove corrupted trials. Then, we ran the runICA algorithm available in EEGLAB v2021.0 (Groppe, Makeig, et al. 2009). Artefactual independent components (ICs) were first identified using the ICLabel toolbox (Pion‐Tonachini et al. 2019) and subsequently checked before their removal.

**FIGURE 1:**
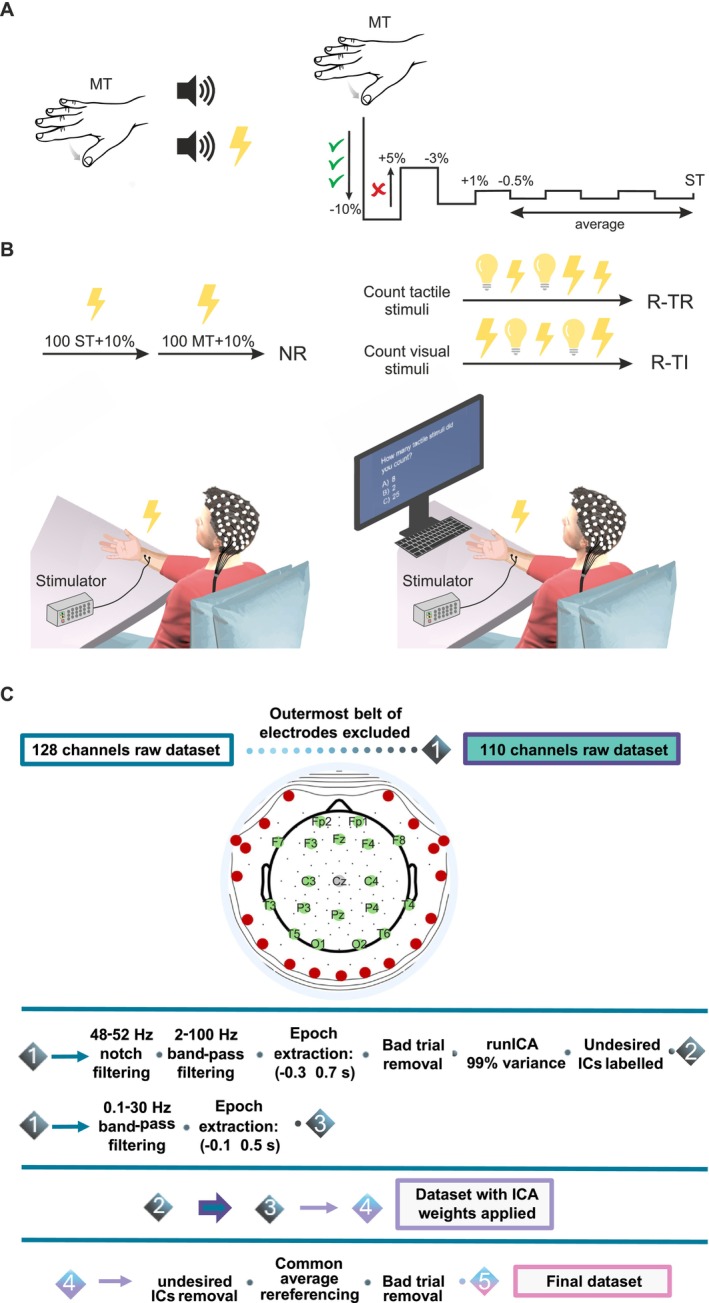
The experimental procedure. (A) The motor threshold was assessed as the minimum amplitude that elicited an observable thumb twitch. To identify the sensory threshold, subjects underwent an adaptive staircase procedure: First, they were asked to listen to two sounds and indicate whether tactile stimulation occurred after the first or second sound. If they identified it correctly three times in a row, the amplitude was reduced by 10% until they made an error or could no longer perceive the stimulation. In such cases, the amplitude was adjusted incrementally, increasing by 5%, and then lowering by 3% after three correct responses. This process continued with finer adjustments (1% and 0.5%) for six cycles, and the somatosensory threshold was calculated by averaging the amplitude levels from these final steps. (B) scalp‐EEG recorded subjects underwent two separate sessions: In the first, they were required to passively perceive two trains of tactile stimuli, delivered respectively at the sensory threshold + 10% and the motor threshold + 10% (NR condition); the second block consisted of 40 trains of unimodal stimuli (tactile or visual), with 20 trains at ST + 10% and 20 at MT + 10%, presented randomly. Participants followed on‐screen instructions to count either visual stimuli (task‐irrelevant, R‐TI) or tactile stimuli (task‐relevant, R‐TR). After each train, three numbers with a letter appeared, and participants pressed the letter corresponding to the correct count. (C) Schematic representation of the complete EEG data preprocessing pipeline, illustrating all major steps from raw signal import to analysis‐ready data. 128‐channel Geodesic Sensor Net. Electrodes shown in red were excluded from analysis. Electrodes in green and white (Cz, reference) represent the 19 channels positioned according to the international 10–20 system.

Separately, the raw EEG dataset was then band‐pass filtered ([0.1, 30] Hz) (Luck 2014) and segmented into epochs time‐locked to the median nerve stimulation ([−0.1, 0.5] ms). The ICA weights obtained earlier were then applied to this dataset, and the previously identified artefact‐related components were removed. Data were re‐referenced with a common‐average reference, and a final rejection of bad trials was carried out through visual inspection to discard trials exhibiting residual artifactual activity (e.g., eye movements and blinks). Figure 1 summarizes the procedure adopted for identifying the individual thresholds (Panel A), the experimental procedure (Panel B), and the preprocessing pipeline (Panel C).

### 2.5 ERP Analysis and Statistics

The ERP analysis was conducted using the Factorial Mass Univariate ERP Toolbox (FMUT) (Fields and Kuperberg 2020). Factorial analyses were performed for each time sample (e.g., every 2 ms) with two within‐subject factors: Amplitude (stimulation at ST + 10% vs. MT + 10%) and Task (No Report, Task‐Relevant Stimulation, Task‐Irrelevant Stimulation). Statistical significance was assessed using a cluster‐based permutation approach (10,000 permutations), with the significance threshold set to 0.01 and an electrode neighbor distance of 4 cm. As an initial control, the same analysis was conducted on the prestimulus baseline (−100 to 0 ms) without baseline correction, to verify the absence of spurious fluctuations. No significant clusters emerged for either the main effects or their interaction. Following this check, trials were baseline‐corrected using the −100‐ to 0‐ms interval, and the same factorial analysis was then applied to the poststimulus window (0–500 ms). Finally, post hoc comparisons were performed using the Mass Univariate ERP toolbox (Groppe, Urbach, et al. 2011), using the same parameters adopted for the previous test. The procedure for data collection, preprocessing, ERP analysis, and statistics is consistent with the approach outlined in Presti et al. (2023).

## 3 Results

We compared SEPs across two stimulation amplitudes (ST + 10% and MT + 10%) and three task conditions (NR, R‐TR, and R‐TI) within the 500‐ms poststimulation window, capturing both early and late components of SEPs. Both amplitude and task influenced the SEP, with a notable interaction between them.

**FIGURE 2:**
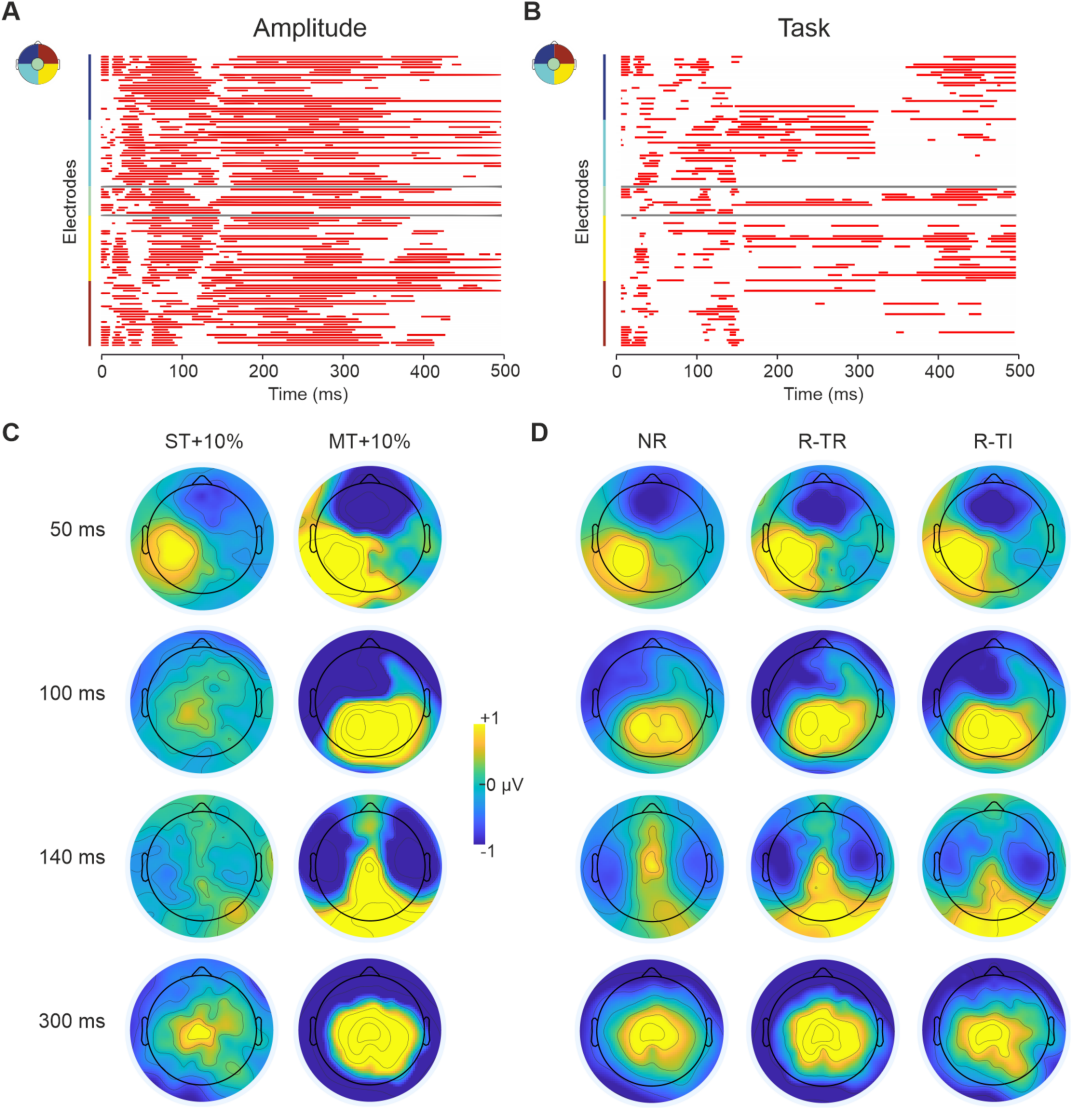
The effect of *Amplitude* and *Task* on SEP modulation. (A) Main effect of *Amplitude* and (B) *Task* on SEP modulation. Time points at which the two amplitude conditions differ significantly are highlighted in red for each electrode. Left‐hemisphere electrodes are arranged from most anterior at the top; right‐hemisphere electrodes from most posterior to most anterior (see inset). (C) Topographical activation following stimulation at ST + 10% (sensory threshold increased by 10%, left column) and MT + 10% (motor threshold increased by 10%, right column) at different temporal instants: 50, 100, 140, and 300 ms (scale: −1 to 1 μV). (D) Topographical activation following stimulation at NR (No‐Report), R‐TR (Report‐Tak Relevant), and R‐TI (Report Task‐Irrelevant) at different temporal instants: 50, 100, 140, and 300 ms (scale: −1 to 1 μV).

Regarding the main effect of *Amplitude*, although the topographical patterns of neural responses appear virtually comparable across different stimulation amplitudes, we found that most channels exhibit pronounced modulation in both the early and late SEP components (*p* = 0.0001, *F*[1,29]). This suggests that variations in stimulus intensity systematically influence the amplitude of sensory evoked potentials, with robust effects visible from the initial stages of processing. In parallel, the *Tas*k factor also emerged as a critical modulator of SEPs (*p* = 0.0001, *F*[2,58]), influencing scalp responses in a temporally extended manner. Even task‐dependent effects became apparent at the initial stages of the stimulus processing, persisting through the entire analyzed time window (Figure 2).

Although stimulus amplitude and task requirements were expected to modulate SEPs (Forschack et al. 2020; Schröder et al. 2021), these factors strongly interacted throughout the SEP time course.

**FIGURE 3:**
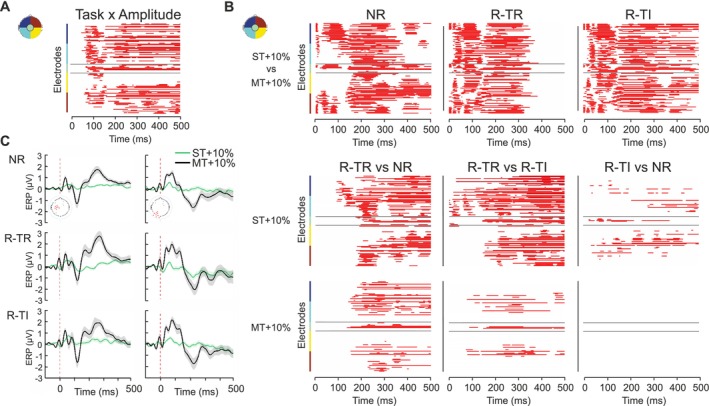
Interaction Task*Amplitude. (A) Interaction Intensity × Task. For each electrode, time points at which conditions differ significantly are highlighted in red. Left‐hemisphere electrodes are arranged from most anterior at the top; right‐hemisphere electrodes from most posterior to most anterior (see inset). (B) Post hoc comparisons between ST + 10% and MT + 10% in the three different experimental conditions. Post hoc comparisons at ST + 10% (upper row) and MT + 10% (lower row) contrasting the three experimental conditions. Time points with significantly higher amplitudes are shown in red. NR: No‐Report, R‐TR: Report Task‐Relevant, R‐TI: Report‐Task‐Irrelevant. Left hemisphere electrodes (from the top) are shown from most anterior, and right hemisphere electrodes are plotted from most posterior to most anterior (see inset). (C) SEP in the six different experimental conditions (green: ST + 10%, black: MT + 10%). SEPs (± SE) averaged across the electrodes indicated in the inset.

As shown in Figure 3, the Task*Amplitude interaction is steadily significant over both early and late SEP components (*p* = 0.0001, *F*[2,58]). We then performed three post hoc comparisons to investigate SEP modulations across the two amplitudes within each task and six other pairwise comparisons between tasks (i.e., NR vs. R‐TR, NR vs. R‐TI, and R‐TR vs. R‐TI) at both ST + 10% and MT + 10%, separately (Figure 3B).

Stimulation intensity has a stronger effect on early SEP components (0–100 ms) in the Report conditions (R‐TI, R‐TR) than in the No‐Report condition, possibly reflecting a greater engagement of the subjects in the task. Conversely, no modulation of late components was found between ST + 10% and MT + 10% stimulations only in the R‐TR condition, indicating that the effect of amplitude is almost abolished when stimuli are task‐relevant (Figure 3B,C). All significant clusters identified in these analyses were associated with *p*‐values < 0.0001 (df = 29).

**FIGURE 4:**
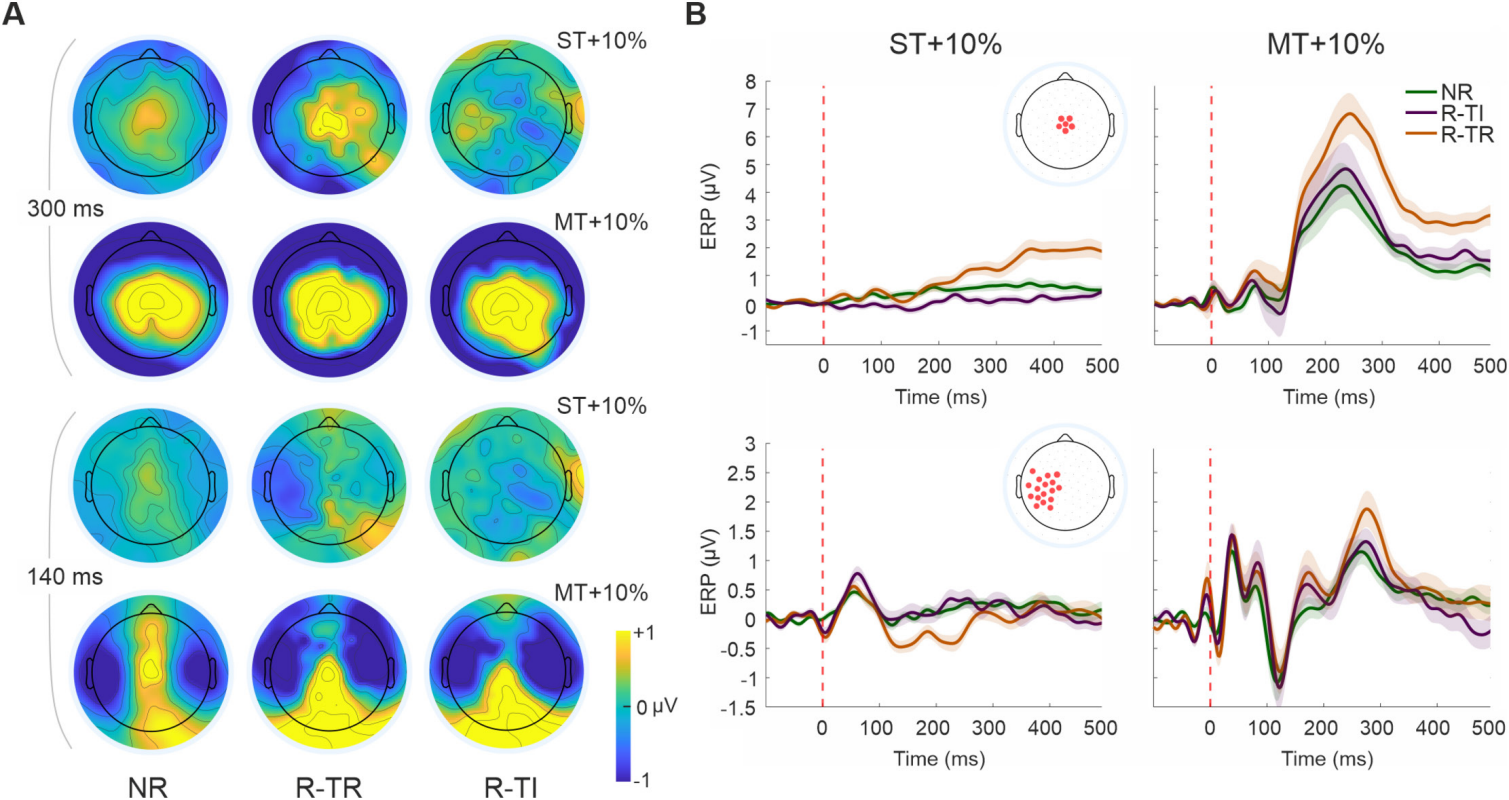
Cluster‐specific topographical activation and SEP waveforms for ST + 10% and MT + 10% stimulation across report conditions. (A) Topographical activation following stimulation at ST + 10% (upper row) and MT + 10% (lower row) in the significant central and left fronto‐central cluster at a range of 300 and 140 ms, respectively (scale: −1 to 1 μV). (B) SEP in the three different experimental conditions (green: NR, orange: R‐TR purple: R‐TI). SEPs (± SE) averaged across the respective cluster of significance (see inset).

Further analysis of within‐amplitude contrasts revealed a consistently significant cluster over central electrodes when contrasting R‐TR against both NR and R‐TI, with the cluster's location and timing aligning closely with the characteristics of the P3b component (time range: 150–500 ms). This finding highlights the distinct role of the report task in engaging neural processes related to conscious tactile perception. More intriguingly, when directly comparing NR with R‐TI, this significant cluster disappeared, suggesting that the no‐report condition functionally resembles a task where participants are explicitly instructed to disregard tactile stimuli (Figures 2B and 4). This suggests that neural processing under NR closely mimics a scenario of intentional stimulus neglect, despite the stimuli being presented well above the detection threshold. Finally, when contrasting NR versus R‐TR at the ST + 10%, we uncover a significant negative left cluster. The centro‐lateral topography and temporal dynamics of this cluster correspond to those typically associated with the N140. Indeed, this component is usually reported as bilateral, albeit with a contralateral predominance (Lindenbaum et al. 2021; Schröder et al. 2021; Auksztulewicz et al. 2012; Forschack et al. 2020). Additionally, we observe an overlap in the temporal range of significance (e.g., 88–200 ms; Figure 4), further reinforcing the idea of an interplay between amplitude modulation and task context.

## 4 Discussion

In this study, we explored how task relevance modulates the SEPs and whether these modulations depend on stimulus intensity. To do this, we designed a paradigm that varied task relevance at three different levels: In the first condition, stimuli were task relevant (R‐TR); in the second, stimuli had to be disregarded, but subjects were nonetheless engaged in a task (R‐TI); in the third condition, subjects were asked to remain passive while perceiving the stimuli (NR). These three conditions were tested across two levels of stimulation amplitude, that is, the individual ST and MT, to determine whether report‐ or task‐related modulations are equally present across perceivable amplitudes or selectively emerge mostly at one of these intensities. Our results demonstrate that SAN is enhanced by a report task, but specifically when the report is task relevant and the stimulation is delivered near the ST. Notably, report responses that are task irrelevant fully overlap with those observed in the NR condition, indicating no distinct modulation. Furthermore, SAN modulations are absent when SEPs are elicited using stimulation at the MT, underscoring its critical dependence on sensory‐level engagement and task relevance.

When examining late SEP components, we observed a different pattern. The amplitude of the P3b was closely associated with the task relevance of the stimulus rather than with the act of reporting. Therefore, our findings support the interpretation of the P3b as a robust marker of task relevance, rather than a correlate of sensory awareness or reporting itself. Meanwhile, SAN appears to be modulated by attentional engagement but only for stimuli near the ST, thus more challenging to detect. Together, these findings posit distinct roles for SAN and P3b in somatosensory conscious perception: SAN reflects sensory‐level attentional processes that are sensitive to stimulus intensity and task engagement, whereas P3b tracks higher level task relevance, independent of ST. This distinction contributes to a more nuanced understanding of how different ERP components map onto key cognitive processes such as awareness, attention, and task relevance.

### 4.1 The Conditional Influence of Task Relevance on Sensory Awareness

Although attention and sensory awareness are usually seen as separate (Lamme 2003), the neuroscience of consciousness continues to explore how they interact (Bola and Doradzińska 2021). In fact, SAN and its counterparts in other sensory modalities, known as PAN, are linked to consciously perceived stimuli—regardless of task relevance—supported by strong electrophysiological evidence (Dembski et al. 2021; Koivisto, Revonsuo, Salminen, et al. 2005; Koivisto, Revonsuo, Lehtonen, et al. 2006; Koivisto, Kainulainen, et al. 2009; Koivisto and Revonsuo 2007). However, task relevance still increases PAN response amplitudes (Dellert et al. 2021; Pitts, Metzler, et al. 2014; Shafto and Pitts 2015; Schelonka et al. 2017; Schlossmacher et al. 2021), challenging the idea of their functional independence from attention.

Our results indicate that the putative underpinnings of somatosensory awareness are not influenced by task relevance when stimuli are delivered well above the detection threshold (i.e., at the MT). However, this is overturned when the same stimuli are delivered near the detection threshold, suggesting that the interaction between attention and sensory awareness may depend on an “on‐demand,” top‐down mechanism. Our results fall between the studies linking task relevance and sensory awareness directly (Doradzińska and Bola 2024) and those that argue for a clear separation between them (Ciupińska et al. 2024; Filimonov et al. 2024). The modulation of negative components occurs only when stimuli are delivered with low salience, possibly sustaining awareness when necessary. This mechanism may explain behavioral results from patients with tactile extinction (Fossataro et al. 2020), where the simultaneous presentation of a visual stimulus alongside a somatosensory one increases the relevance of the latter, thereby facilitating the recovery of impaired detection abilities.

### 4.2 P3b: The Marker for Task Relevance

Over the years, the conception about the functional role of P3b has gradually shifted from the neural correlate of sensory awareness to a major player in postperceptual processes, such as working memory, stimulus categorization, and reporting (Cohen et al. 2020; Pitts, Padwal, et al. 2014; see also Dembski et al. 2021). Recently, growing evidence highlights a direct link between task relevance and the P3b component, consistent with findings from classical inattentional blindness paradigms. In these, unexpected stimuli go unnoticed because of attention being focused on a primary task and elicit P3b with lower amplitude compared with attended stimuli (Pitts, Metzler, et al. 2014; Pitts, Martínez, et al. 2012). Schröder et al. (2021) found that P3b correlated with conscious perception only when stimulus detection was the primary task but not in a tactile–visual matching task where both the presence and absence of the stimulus were equally task‐relevant. Our results further support this evidence and add to the debate about the functional role of P3b in reflecting conscious perception, strictly linking it with task relevance rather than other postperceptual processes. In particular, by isolating task relevance as the only factor under examination (i.e., stimuli are always delivered at the verge of detection), we found that task‐irrelevant stimuli elicit a P3b comparable with a no‐report train of passive stimuli, even if subjects are actively engaged in a task requiring working memory and report production. Notably, a P3b is still elicited when stimuli are passively perceived (NR condition, both amplitudes), and it is only modulated by task relevance, suggesting it may subserve another function in the somatosensory domain. The presence of a late component following somatosensory stimulation has been reported in seminal SEP studies (Desmedt and Robertson 1977; Desmedt et al. 1977; Hämäläinen et al. 1990; Michie et al. 1987; Zopf et al. 2004). However, its functional role has not been thoroughly investigated. We hypothesize that, despite not directly reflecting phenomenal awareness, the consistent modulation driven by stimulus amplitude suggests it may serve as a marker of sensory transformation. In this process, information becomes accessible to a broader, distributed network of cortical regions (Mashour et al. 2020).

## Author Contributions

**Alice Giorgi:** conceptualization, data curation, formal analysis, investigation, methodology, writing – original draft, writing – review and editing. **Erica Bonomi:** investigation, writing – review and editing. **Enrico Salemi:** investigation. **Davide Albertini:** visualization, writing – original draft, writing – review and editing. **Flavia Maria Zauli:** visualization, writing – review and editing. **Ezequiel Pablo Mikulan:** writing – original draft, writing – review and editing. **Andrea Pigorini:** writing – original draft, writing – review and editing. **Pietro Avanzini:** conceptualization, writing – original draft, writing – review and editing. **Maria Del Vecchio:** conceptualization, formal analysis, funding acquisition, investigation, project administration, supervision, writing – original draft, writing – review and editing.

## Conflicts of Interest

The authors declare no conflicts of interest.

## Peer Review

The peer review history for this article is available at https://www.webofscience.com/api/gateway/wos/peer‐review/10.1111/ejn.70262.

## Data Availability

The EEG preprocessed and raw data have been uploaded as unique zip files in a public repository on OSF (https://osf.io/hqkym/files/osfstorage).
